# Next-Generation Sequencing Strategies During the 2024–2025 Avian Influenza A(H5N1) Emergency Response in the U.S

**DOI:** 10.3390/v18040482

**Published:** 2026-04-21

**Authors:** Julia C. Frederick, Kristine A. Lacek, Matthew J. Wersebe, Bo Shu, Lisa M. Keong, Juliana DaSilva, Malania M. Wilson, Sydney R. Sheffield, Jimma Liddell, Natasha Burnett, Reina Chau, Amanda H. Sullivan, Yunho Jang, Juan A. De La Cruz, Elizabeth A. Pusch, Dan Cui, Yasuko Hatta, Sabrina Schatzman, Norman Hassell, Xiao-Yu Zheng, Ha T. Nguyen, Larisa Gubareva, Rebecca Kondor, Han Di, Vivien G. Dugan, Charles T. Davis, Benjamin L. Rambo-Martin, Marie K. Kirby

**Affiliations:** Influenza Division, National Center for Immunizations and Respiratory Diseases, US Centers for Disease Control and Prevention, Atlanta, GA 30329, USA

**Keywords:** next-generation sequencing, avian influenza, H5N1, public health, response structure

## Abstract

The first influenza A(H5N1) human case associated with the A(H5N1) dairy cattle outbreak in the United States was identified in April 2024. The U.S. CDC response to this outbreak was activated days later and remained active until July 2025. During this time, 70 human cases of influenza A(H5N1) were detected with a range of epidemiological links to sources of exposure. Next-generation sequencing (NGS) of human samples was an effectual mechanism for tracking and analyzing the outbreak evolution throughout the response. Due to the specimens’ importance and their variable physical quality, an assortment of laboratory methods was utilized including influenza segment-specific amplification, enrichment capture, short-read, and long-read sequencing. Combining these methods allowed for high-quality genomic data production with rapid turnaround times—typically 2 days from sample receipt to public database submission. By leveraging replicate sequencing, enrichment capture, and sequencing of diagnostic amplicons, valuable genomic data could be produced directly from human clinical specimens that would have normally been considered too weak for routine virologic surveillance sequencing. The resulting assemblies were characterized and analyzed by CDC and shared with local and state public health authorities to facilitate case investigations and risk assessment. These data were further used for phylogenetic analyses of viruses from human cases to investigate likely animal-to-human transmission events and identify clusters within the outbreak that might indicate trends in the types of exposures. Through the adaptable laboratory workflow and the rapid release of viral genomic data, the public health risk mitigation strategies could be evaluated and adjusted in real time.

## 1. Introduction

Highly pathogenic avian influenza (HPAI) A(H5N1) virus, which is globally established in a wide range of hosts, has been a continuous public health threat for decades [1]. The United States had previously seen HPAI outbreaks in domestic poultry, but in March 2024, A(H5N1) clade 2.3.4.4b genotype B3.13 virus was detected circulating in dairy cattle [2]. The first detection of a human case associated with the dairy cattle outbreak was identified in early April 2024, and the U.S. Centers for Disease Control and Prevention (CDC) avian influenza A(H5N1) emergency response was activated on 4 April 2024. During the response period, a distinct genotype, HPAI A(H5N1) clade 2.3.4.4b D1.1, was subsequently detected in dairy cattle in January 2025, following frequent detection in wild birds and sporadic outbreaks in poultry [3,4].

The CDC A(H5N1) response was operational from April 2024 to July 2025, and during that time, there were 70 PCR-confirmed human cases of A(H5N1) in the United States. Forty-one cases were caused by cattle exposure, 24 through poultry exposure, two by other animal exposure (one resulting in death), and three cases through an unknown exposure source [5,6]. Six cases were detected via national influenza surveillance, while the other 64 were detected through targeted A(H5N1) surveillance [6].

Specimens received by the CDC were original clinical specimens from patients with a range of disease severity and clinical presentations. Many of these specimens had high Ct values, near the threshold for detecting influenza A virus diagnostically, and notably higher than typical surveillance sequencing limits of detection. The significance of each case warranted additional sequencing and bioinformatic strategies for low viral load clinical specimens that failed CDC’s standard influenza genomic surveillance pipeline but were still positive for A(H5). Throughout the emergency response, multiple next-generation sequencing (NGS) techniques were utilized to produce as much informative data as possible about each case of A(H5N1) as rapidly as possible. These included CDC’s M-RTPCR [7], enrichment capture techniques, and the development of targeted hemagglutinin (HA) and neuraminidase (NA) primers for HPAI A(H5N1). Multiple libraries prepared with different methods were then sequenced on both Oxford Nanopore Technologies (ONT) sequencing for long reads and rapid data generation, as well as deep and replicate sequencing of short reads with Illumina.

The 2024–2025 Avian Influenza A(H5N1) emergency response in the U.S. required public health scientists across disciplines to rapidly adapt methodologies and analyze data in real-time to continuously inform public health risk mitigation strategies. The adaptations and analyses performed over the course of the response are described here to demonstrate how a surveillance sequencing workflow can be adjusted to respond to an influenza outbreak. Due to rapid laboratory work and real-time analyses, CDC has diagnosed, characterized, and reported on these cases with high confidence, providing valuable detailed data to public health authorities throughout the United States during this response. The genomic characterization of these cases, together with genomic data from animal viruses published by the U.S. Department of Agriculture (USDA) National Veterinary Services Laboratory (NVSL), continues to inform diagnostics, therapeutics, and pandemic preparedness nationwide.

## 2. Materials and Methods

### 2.1. Sample Acquisition

The majority of cases were identified through targeted epidemiological tracking of farm workers and other reported infected animal exposures. Importantly, additional cases were identified through national influenza surveillance [3]. The original clinical specimens collected during the A(H5N1) emergency response were rapidly shipped by local and state public health laboratories to CDC for confirmatory testing and additional virologic characterization [3].

### 2.2. RNA Extraction and Amplification

The isolation of nucleic acids for confirmatory testing, subtyping, and sequencing was done using a QIAmp viral RNA purification kit (Qiagen, Hilden, Germany) following the manufacturer’s instructions, with 120 µL of clinical specimen as input, when available. The samples that were diagnostically confirmed positive for influenza A and H5 targets were then processed for sequencing [8,9]. Positivity is determined by samples having Ct values of <38 for InfA, H5a, or H5b.

Compared to seasonal influenza cases, which are derived from respiratory specimens, the clinical specimens associated with this response were predominantly conjunctival swabs and had higher Ct values—requiring a multi-target amplification approach. Whole-genome M-RTPCR amplification of extracted RNA was performed in quadruplicate when volume permitted using Super-Script III One-Step RT-PCR with Platinum Taq High Fidelity (Invitrogen, Carlsbad, CA, USA). The amplification was conducted with the universal influenza A primer set as described in Zhou et al. 2009, which binds to the untranslated region of all influenza A viral genome segments [7].

Targeted amplification of the HA and NA segments was completed using four sets of region-specific primers to increase the sequencing yield of specimens with low viral titer. Each specimen was amplified in individual 25 µL reactions using the following primer pairs: H5_HA_F23M and H5_HA_R1000m; H5_HA_F810Y and H5_HA_R1773; H5N1_NA_F18 and H5N1_NA_R856; and H5N1_NA_F638 and H5N1_NA_R1444 (Table 1). These primer pairs were developed during the A(H5N1) response and were the most used scheme. Additional primer pairs used during the response are listed in Supplemental Table S1.

### 2.3. Genome Sequencing

Amplicons were purified using exonuclease I (Thermo Fisher Scientific, Waltham, MA, USA), and amplicon quality and size were assessed using QIAxcel Advanced (Qiagen, Hilden, Germany). Library preparation would proceed if the negative template controls and positive template controls passed quality control (QC; i.e., negative controls with no amplification in the desired size range and positive controls with visible amplification in the desired size range). For all sequencing methods, the whole-genome amplicon replicates were processed individually. Prior to library generation, the following sample amplicon pools were made: HA-targeted amplicons, NA-targeted amplicons, and a pool of both whole-genome and targeted amplicons.

Oxford Nanopore Technologies libraries were generated following manufacturers’ protocol for Native Barcode chemistry (SQK-NBD114.96; Oxford Nanopore Technologies, Oxford, UK). Libraries were checked for size distribution, and they were pooled and diluted to 8–80 ng. The final pool was then loaded onto GridION using either a R10.4.1 flow cell (FLO-MIN114; Oxford Nanopore Technologies, Oxford, UK) or Flongle flow cell (FLO-FLG114; Oxford Nanopore Technologies, Oxford, UK) with the Flongle adapter. The on-board GridION MinKNOW software v24.02.10 was set for a 12 h run time, with live super accurate (SUP) base calling enabled, barcoding enabled with required dual-end barcodes and barcode trimming, read Q-score filtered to >10, and minimum read length of 200 bp.

The Illumina libraries were generated using quarter-volume reactions of the Illumina DNA Prep Kit (Illumina, San Diego, CA, USA). The libraries were individually barcoded and pooled to 2 nM final concentration based on average library size, and the concentration was quantified on a Qubit using the 1× dsHigh Sensitivity Kit (Invitrogen, Carlsbad, CA, USA). The final pool was loaded on Illumina MiSeq using a 300-cycle v2 kit (Illumina, San Diego, CA, USA) with the run set for 150 base paired-end sequencing. The libraries for ONT and Illumina sequencing were processed simultaneously for all cases associated with a new or unknown source of exposure, including any H5-infected animal exposures in new locations; subsequent cases from the same location/source of animal exposure were only sequenced using Illumina methods.

The samples from cases with an unknown source of exposure or patients experiencing severe illness (*n* = 4) were further sequenced using enrichment capture. Double-stranded cDNA was generated from sample RNA following the manufacture’s protocol for Total Nucleic Acids Library Preparation EF Kit 2.0 for Viral Pathogen Detection and Characterization (Twist Bioscience, South San Francisco, CA, USA). After cDNA generation, the previously generated amplicons were also included in the workflow for enrichment. Following the manufacturer’s protocol, the cDNA and amplicons were incubated for 15 min at 37 °C for fragmentation, individually indexed libraries were generated, and then pools were made based on library size and concentration. Target-enriched library pools were prepared following Target Enrichment Fast Hybridization protocol using the Comprehensive Viral Panel probe set (Twist Bioscience, South San Francisco, CA, USA). The following modifications were made to the protocol due to the interest in a single pathogen and the low viral loads of the samples: universal blockers and blocker solution were increased (12 µL and 7.5 µL, respectively), the hybridization reaction was set for 2.5 h at 60 °C, and 10 cycles for post-capture PCR amplification were carried out. A final loading pool at 2 nM was prepared and loaded onto Illumina MiSeq using a 300-cycle v2 kit, with the run set for 150 base paired-end sequencing.

### 2.4. Oxford Nanopore Sequencing Analysis

Assembly and annotation of ONT sequencing used MIRA-NF [10] with the Flu-ONT module which requires 50× median coverage, 90% gene segment completeness, and fewer than 10 minor single-nucleotide variants (SNVs) exceeding 5% minor allele frequency (MAF) for a gene segment to pass quality control. Individual bases require an average 30× coverage and ≥10 Phred score to call. Premature stop codons were not permitted in the HA and NA gene segments.

**Figure 1:**
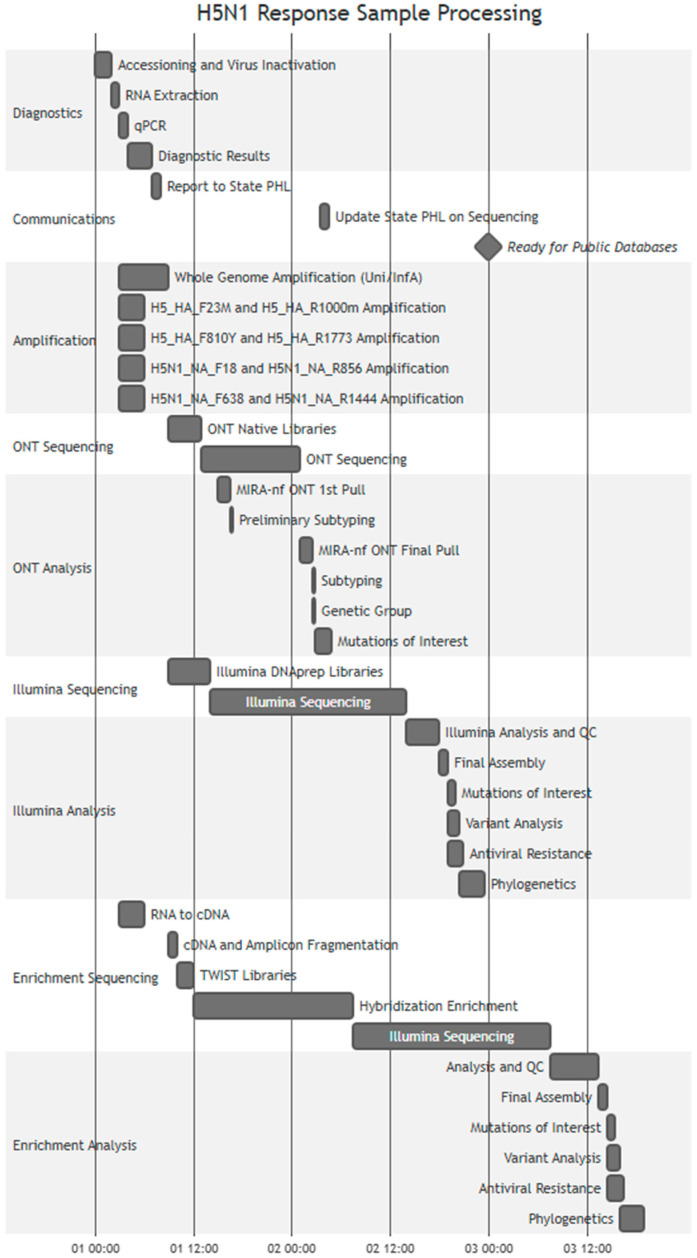
Timeline of CDC’s 2024–2025 influenza A(H5N1) Emergency Response in the U.S. sample processing. Activity timing (displayed as Day Hour:Minute on axis) is determined by typical work done throughout response by responders. Any wait step times are encompassed within the overall time bar for that activity.

Due to the real-time basecalling and demultiplexing of ONT, the data were available within 1 h of sequencing, allowing initial assemblies to be generated, which reflected HA and NA subtype and partial genomes. To maximize coverage, read pools were made per specimen by combining demultiplexed FASTQs of molecular replicates and targeted sequencing primers. The assembly data for the HA and NA gene sequences were generally available for further analysis within the same business day (Figure 1).

### 2.5. Illumina Sequencing Analysis

Illumina sequencing data were demultiplexed using bcl2fastq v2.20.0 from the nf-core demultiplex module v1.4.0 [11,12] with the following additional parameters: no lane splitting and 0 barcode base mismatches allowed. All molecular replicates were analyzed individually to identify any introduced errors, and then read pools were created for each specimen to increase coverage.

Consensus assemblies and variant calling per sample were completed using the FLU-utr module in IRMA v1.2.0 [13]. Gene segment assemblies were then analyzed for quality and mixed infections using the following metrics: average segment coverage ≥100×; coverage ≥25× per base in coding regions and ≥20× at unaligned terminal edges; identifying stop codon presence; minor variants per gene segment do not exceed 5 sites with ≥5% MAF; mixed sites (≥20% MAF) do not exceed 10% of segment sites; within 15 consecutive bases there are not ≥3 mixed bases. Some gene segments did not have complete assemblies that met the per-base coverage requirements. In these cases, the segment assembly would have non-passing bases masked with N to only include the region(s) that passed all other quality metrics. The partial gene segment assemblies were used in downstream analysis.

### 2.6. Comparative Analysis of Assemblies

Quality-passing assemblies were aligned to A(H5N1) structural genome references to check for unknown insertions or deletions using MUSCLE v3.8.31 [14]. Any new indels were confirmed through further sequencing attempts of cell- or egg-propagated virus isolates. Variant sites required a coverage depth of at least 100× to be identified. Mixed-base sites were identified in the final consensus assembly if the MAF was greater than 0.20, while minor variants needed to have a MAF of ≥0.02 to be considered for the analysis. A minor variant was called within a specimen if there were more than 2 replicates that had the gene segment assembled and >65% of replicates had a variant called at that site, allowing for the identification and removal of PCR-induced errors from analysis. Substitutions in NA and PA gene segments were compared against aggregated tables of genetic markers associated with reduced antiviral susceptibility [15,16]. An additional primer trimming step using bbduk v39.01 was taken in an attempt to remedy variants observed in and near the primer-binding regions (mm = f hdist = 1 ordered = t minlength = 0 k = 11 mink = 6 restrictleft = 35 rcomp = t) [17].

### 2.7. External Data Acquisition

The USDA-NVSL uploaded influenza A(H5) virus sequence data from cattle, wild birds, peridomestic animals, and other hosts to NCBI’s Sequence Read Archive (SRA). Additional submissions were also made to NCBI’s GenBank database after epidemiological investigations were resolved or appropriate notifications were given. An informatics workflow, leveraging the R package ‘rentrez’ v1.2.4 [18], was created to make queries to both SRA and GenBank that extracted metadata from the NCBI uploads and to combine it with the SRA metadata for completeness. After extracting the metadata, missing and incomplete metadata was coalesced across sources to provide information on the reported collection date and location, and eliminate duplication in the data used for subsequent analyses [19]. Next, sra-tools v3.2.1 [20] was used to download the FASTQs associated with the USDA sample submissions, and these samples’ NGS data were processed with the MIRA-NF Flu-Illumina module.

In addition to the data downloaded from NCBI, the Global Initiative for Sharing All Influenza Data (GISAID) EpiFLU^TM^ database was searched for sequences closely related to the ongoing influenza A(H5) outbreaks. Using A/Texas/37/2024 and A/Washington/255/2024 as representative genomes for the influenza A(H5N1) B3.13 and D1.1 genotypes, respectively, all available gene segment nucleotide sequences were aligned and a pairwise TN93 genetic distance was calculated between the reference and all queried strains [21]. The inclusion of sequences was limited to distances of less than 0.10 compared to the genotype references or to HA sequences labeled as clade 2.3.4.4b. Since many sequences released by USDA on SRA were subsequently assembled and deposited in EpiFLU by GISAID, sequences were de-duplicated by their unique isolate IDs available from both sources. The sequences from the CDC, USDA, and GISAID were aggregated into a single unified database for subsequent analyses.

### 2.8. Evolutionary Analysis

The USDA has defined several different “genotypes” of clade 2.3.4.4b A(H5Nx) viruses co-circulating in the Americas. Each genotype reflects a unique constellation of gene segments with distinct genetic backgrounds, including segments from Eurasian HPAI A(H5) virus (denoted “ea”) introductions to the Americas and segments derived from circulating American lineage low pathogenic avian influenza (LPAI) viruses (denoted “am”). All retained segment sequences were characterized by GenoFLU [2] to determine the appropriate genotype in the case of complete genomes or to record the gene segment lineage in the case of partial genomes.

The evolutionary relationships among human cases and animal samples were inferred by time-scaled phylogenies within a maximum likelihood framework using the phylodynamic tool kit available in treetime v0.11.4 [22]. Initial phylogenetic trees per gene segment were inferred using the Maximum Parsimonious Likelihood Estimation method as implemented in CMAPLE v1.1.0 [23]. Trees were passed to treetime, as implemented in the augur workflow v29.0.0 [24], which output the phylodynamic analyses in Nextstrain’s Auspice format for easier exploration [25]. Samples with questionable metadata or apparent issues in sequencing quality were removed from analyses to improve the topology of the inferred phylogenetic trees.

## 3. Results

**Figure 2:**
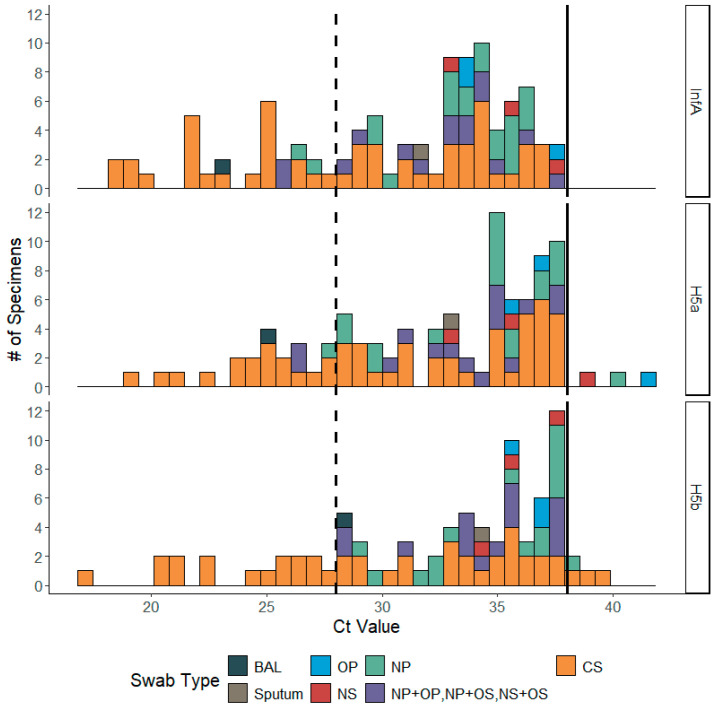
Distribution of diagnostic Ct values of positive specimens received during the CDC’s 2024–2025 influenza A(H5N1) emergency response in the U.S. The diagnostic tests are for influenza A (**top** panel), and two different H5 targets (**middle** and **bottom** panels). The LOD for the diagnostic assay is less than or equal to a Ct of 38 to be determined positive (solid line). The LOD for high throughput seasonal surveillance sequencing is less than or equal to a Ct of 28 (dashed line).

Across the 70 human cases of avian influenza A(H5), 105 specimens were sequenced, including 17 cases with multiple positive swab types, three specimens from follow-up sampling, and six propagated viral isolates from original clinical specimens that could not be sequenced. The original clinical specimens were collected from either conjunctival swab (CS; n = 58; average InfA Ct = 28.8; median InfA Ct = 29.3), nasopharyngeal swab (NP; n = 22; average InfA Ct = 33.3; median InfA Ct = 34.2), nasal swab (NS; n = 3; average InfA Ct = 35.4; median InfA Ct = 35.6), oropharyngeal swab (OP; n = 3; average InfA Ct = 35.1; median InfA Ct = 33.8), bronchoalveolar lavage sample (BAL; n = 1; InfA Ct = 23.4), or sputum sample (n = 1; InfA Ct = 31.7). Additionally, 17 specimens with combined swab types were also submitted (NP + OP, NP + OS, or NS + OS; average InfA Ct = 32.1; median InfA Ct = 33.2) (Figure 2; Supplemental Table S3).

Across sequenced specimens, there were five CS specimens with InfA Ct values below 20 and all assembled full genomes; 23 specimens of varying swab types had InfA Ct values between 20 and 28, averaging 7.6 gene segments assembled per sample; all other specimens had InfA Ct values greater than 28, averaging 2.6 gene segments assembled. The genetic data from 59 specimens representing 54 cases (77.1%), which met quality standards and gene segment completeness, were released to public databases (Supplement Table S2). The remaining specimens’ genetic data contained low levels of influenza sequences resulting in low quality assemblies or had fewer gene segments completed when compared to publicly released sequence data from the same case. Summary information of the number of cases to number of publicly released genomes can be found in Supplemental Figure S1.

### 3.1. Consensus Sequence Comparisons

There were 101 ONT replicates, averaging 153,595 reads per replicate with an average of 23.74% reads mapping to influenza A genome. Additionally, 351 Illumina replicates were sequenced, averaging 1,190,500 reads with 35.78% mapping to influenza A genome. The read pools of all replicates by sample and platform averaged 597,234 reads (20.93% influenza mapping, minimum = 0.02%, maximum = 76.69%) for ONT, and 6,915,555 reads (44.07% influenza mapping, minimum = 0.01%, maximum = 99.18%) for Illumina. By seasonal influenza virus surveillance threshold standards, a sample is considered to have low influenza viral sequence content if less than 1.5% of reads map to an influenza genome. Read pool analyses indicated low influenza viral sequence content by Illumina for 62 samples and for 27 samples through ONT.

Illumina replicates had 80% of segment assemblies identical; for those with discrepancies, there was an average hamming distance of 2.74 (a pairwise comparison of equal-length sequences counting the number of positions where bases are not exact matches). Most of the discrepancies occurred in the HA and NA gene segments, largely due to inconsistent mixed-base calls at primer-binding sites. The ONT replicates had 86% of segments identical with an average hamming distance of 2 among segments with discrepant bases. For cases with multiple positive swab types, consensus sequences were also analyzed for similarity. Of the 17 cases with multiple swab types, 14 cases had at least one gene segment assembly overlapping with the paired swab. There were 42 paired gene segments in total, and 16 pairs had identical sequences; the others had an average hamming distance of 1.2.

For the consensus genomes from read pool analyses, Illumina was more likely to be complete with ONT assemblies having fewer gene segments passing all QC metrics typically due to indels or low coverage of segment terminal ends. Of the 28 samples that were sequenced on both Illumina and ONT platforms, 18 had at least one QC-passing gene segment assembly from read pools on both platforms, with 72% of segment assemblies identical across platforms. Of those that had site discrepancies, there were eight mixed sites in an Illumina assembly where the ONT assembly had a nucleotide-specific call, while there was one site that was a mixed-base call for ONT and a nucleotide-specific call for Illumina. In all cases, the mixed-base included the specific nucleotide of the other assembly. There was also one site for one sample that had a discrepant base call in the merged read assemblies at site 15 of the HA assembly (Illumina A; ONT G), which was within the primer-binding region of primer H5_HA_F23M.

### 3.2. Minor Variant and Mixed Site Analysis

The 10 most prevalent variant sites in consensus sequences are in a primer-binding region on either the HA or NA gene segment, with an average MAF of 0.14 (minimum = 0.04; maximum = 0.24). Primer trimming prior to assembly removed a three-base artificial deletion that was infrequently seen in the HA consensus right after the H5_HA_F23M binding site (bases 21–23) and slightly decreased the MAF to 0.12 for the variants within the primer-binding regions.

**Figure 3:**
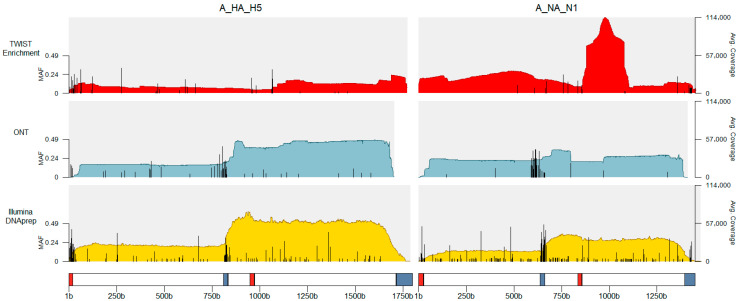
Variant sites and the minimum allele frequencies across HA and NA open reading frames for replicate samples. On the segment diagram, red boxes indicate the binding location for one primer pair for HA or NA, and blue boxes indicate the binding location for the secondary primer pair. Bases are called as minor variants if MAF is >0.02, and as mixed bases if the MAF > 0.2.

After removing all variants that fall within the primer-binding sites of HA and NA, there were 50 specimens (216 replicates) that had QC-passing minor variants and 899 variant sites across all gene segments and samples. When accounting for the replicate thresholds needed to call a variant, the number of specimens was reduced to 28 with 163 replicates and 808 variant sites. There were 476 variant sites present in Illumina-sequenced replicates, 311 sites in ONT-sequenced replicates, and 30 variant sites sequenced on both instruments (Figure 3).

### 3.3. Sequencing of Diagnostic Amplicons and Enriched Libraries

For cases with an unknown source of exposure where the original specimens were close to the diagnostic limit of detection and for cases experiencing severe illness, original specimens were subjected to targeted sequencing approaches. The 200 bp amplicons produced from the CDC H5 diagnostic assay for two cases were sequenced using ONT to compare the amplicon sequence to the control H5 materials, which ruled out the contamination of the specimens, confirming a true positive result.

There were three cases that were sequenced using targeted enrichment: A/Missouri/121/2024 (TWIST Respiratory Virus Panel), A/California/192/2024 (TWIST Comprehensive Viral Panel), and A/Louisiana/12/2024 (NP swab, and NP + OP swab; TWIST Comprehensive Viral Panel). The sequences from A/Louisiana/12/2024 after amplification and enrichment were assembled into a complete genome. For the NP + OP swab type, 53.3% of sequences were mapped to influenza, while NP swabs yielded 15.2%. The enrichment sequencing attempts of A/Missouri/121/2024 and A/California/192/2024 did not assemble into full genomes and had influenza mapping of 0.01% and 3.1%, respectively. However, all specimens sequenced from the enriched libraries had enough of the HA and NA gene segments assembled to have the case confirmed as an A(H5N1) subtype assigned to the 2.3.4.4b clade. Assemblies generated from enrichment had a higher rate of variant calls, with 84 unique variant sites (21 sites/sample compared to Illumina DNAprep 10 sites/sample) and 14 mixed-base calls (3.5 sites/sample compared to Illumina DNAprep 0.89 sites/sample; Figure 3).

### 3.4. Phylogeny of Viruses Identified in Human Samples

**Figure 4:**
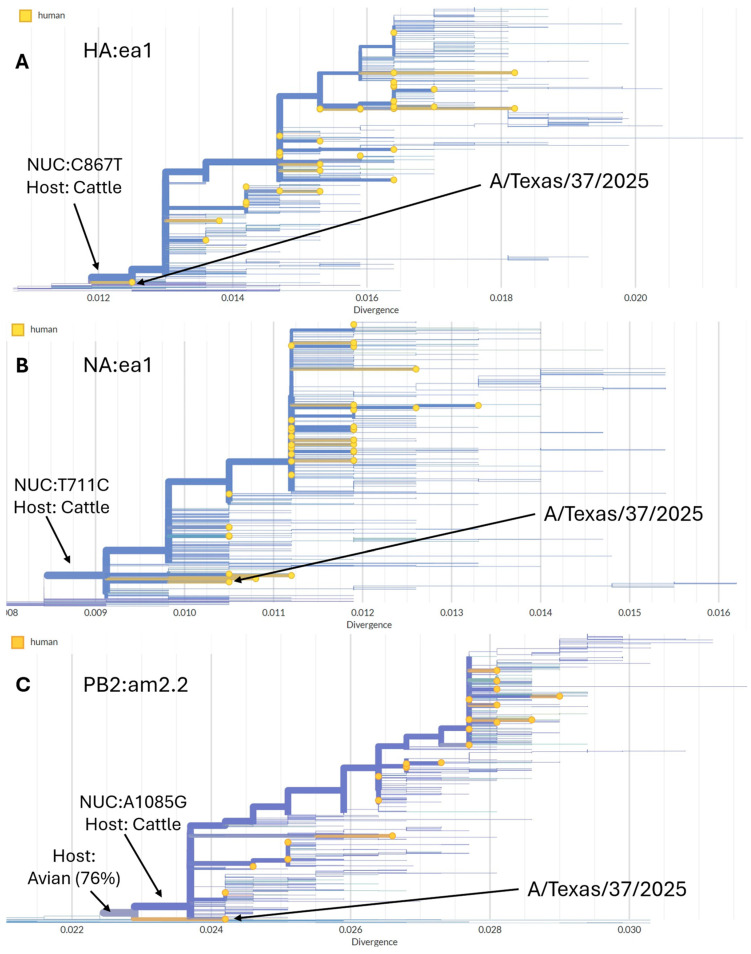
Maximum likelihood phylogenetic trees of B3.13 gene segments with branch lengths in units of divergence (average number of nucleotide substitutions per site). Node and branch colors are depicted by host where yellow is human and blue is dairy cattle. (**A**) HA (segment lineage ea1) gene tree showing dairy cattle outbreak clade; all B3.13 HA segments descend from an ancestral virus similar to A/Ross’s Goose/Kansas/W23-930F/2023 (EPI_ISL_19064371; Genotype Minor60) collected in December 2023. (**B**) NA (segment lineage ea1) gene tree showing the dairy cattle outbreak clade descending from an ancestral virus similar to A/turkey/Minnesota/23-031508-001-original/2023 (EPI_ISL_18695300; genotype B3.6) collected in October 2023. (**C**) PB2 (segment lineage am2.2) gene tree showing the dairy cattle outbreak clade descending from A/green-winged teal/Montana/23-030444-003-original/2023 (EPI_ISL_19660858; H6N8) collected in September 2023. Like in the HA gene tree, A/Texas/37/2024 is outside the dairy cattle clade and sits on a branch defined by unique nucleotide mutations. However, it is descended from the same ancestral population. All USDA dairy cattle sequences and subsequent human cases are within a single monophyletic clade.

All human cases of A(H5N1) identified in the US were from clade 2.3.4.4b and belonged to three separate genotypes—B3.13, D1.1, and D1.3. Inferring gene segment lineage-specific phylogenetic trees allowed the visualization of the evolutionary relationships among human infections and those occurring in animal hosts. For the B3.13 HA (lineage ea1), human cases are nested within the larger dairy cattle-specific outbreak (Figure 4A). This pattern is recapitulated in gene trees derived from the NA (ea1) and PB2 (am2.2) sequences as well (Figure 4B,C). However, the topology at the start of the dairy cattle outbreaks is difficult to resolve due to limited data immediately after the spillover. All USDA sequences of genotype B3.13 from dairy cattle and CDC sequences from human infections are within a single monophyletic clade defined by the homoplastic nucleotide mutation at C867T, except for the first human case, A/Texas/37/2024 (EPI_ISL_19027114), which sits outside the main dairy cattle clade. The NA gene tree shows that all B3.13 NA sequences belong to a single monophyletic clade defined by a single homoplastic nucleotide mutation at T711C. The PB2 gene segment tree shows A/Texas/37/2024 outside the cattle clade, like in the HA tree, and sitting on a branch defined by unique nucleotide mutations at sites C948T and A1962G, along with the homoplasy at site G1879A (leading to the PB2:E672K amino acid substitution known to confer mammalian adaptation). However, it is still sampled from the same ancestral population as the cattle sequences. All USDA PB2 sequences from dairy cattle and subsequent sequences from human cases are within a single monophyletic clade defined by the unique nucleotide mutation at site A1085G (leading to the amino acid substitution at PB2:E362G) and the homoplastic mutation at site A1891C causing the amino acid substitution at M631L.

**Figure 5:**
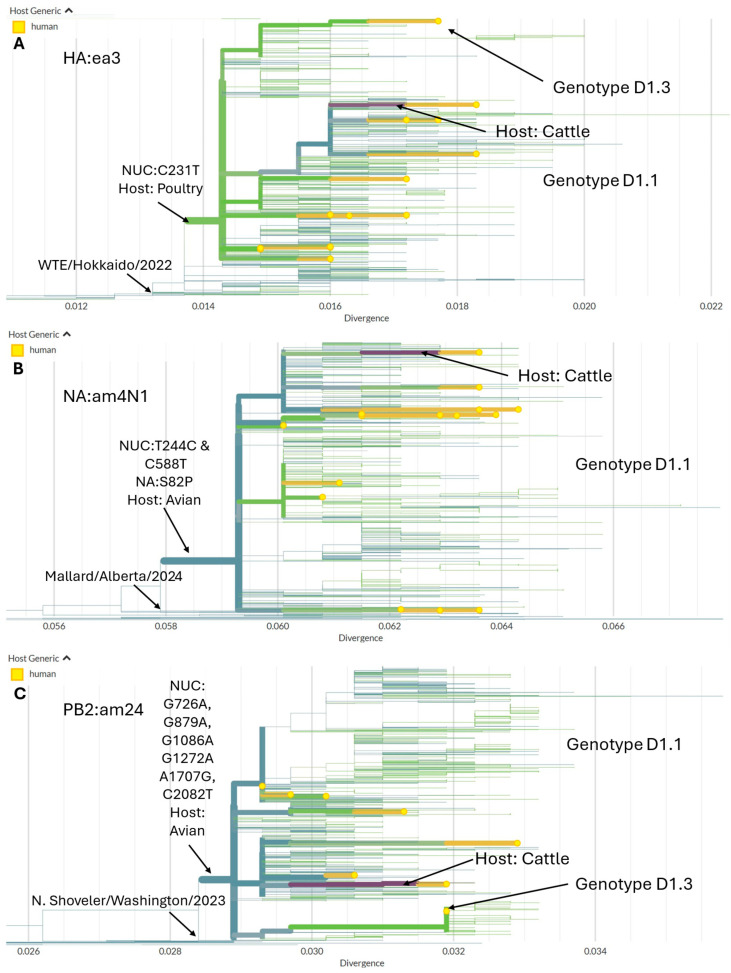
Maximum likelihood phylogenetic trees of D1.1 and D1.3 gene segments with branch lengths in units of divergence (average number of nucleotide substitutions per site). Human sequences are in yellow, poultry in green, avian/wild birds in teal, and cattle in purple. Arrow indicates the location of the D1.3 genotype sequence. (**A**) HA (segment lineage ea3) gene tree showing the 2024–2025 outbreak clade descended from a virus related to A/white-tailed eagle/Hokkaido/20221123001/2022 (EPI_ISL_16955829; Genotype A3) collected in November 2022. Human samples are nested within larger zoonotic outbreaks. (**B**) NA (segment lineage am4N1) gene tree showing the outbreak clade and primarily was associated with LPAI viruses circulating among North American migratory waterfowl. Am4N1 was poorly sampled in the time leading up to the outbreak but likely descends from viruses related to A/Mallard/Alberta/69/2024 (EPI_ISL_19713494, H1N1) collected in August 2024. (**C**) PB2 (segment lineage am24) gene tree showing the outbreak clade descending from a population that includes gene sequences related to A/Northern shoveler/Washington/23-038316-011-original/2023 (EPI_ISL_19559130; LPAI H5N2).

For the D1.1 and D1.3 HA (lineage ea3) sequences, human cases are nested within the larger wild bird- and poultry-associated clade (Figure 5A). The HA gene segment tree shows the recurrent transitions from wild birds to poultry (teal to green branches) and from wild birds to dairy cattle (teal to purple). Human samples are nested within groups of data representing larger animal outbreaks (yellow). Early detections of ea3 HA sequences were primarily along the Pacific coast. The 2024–2025 outbreak consists of a single monophyletic clade defined by the homoplastic nucleotide mutation at position C231T, which has spread across the entire continental US. The NA gene tree shows that the D.1.1 outbreak clade was primarily associated with LPAI viruses circulating among North American migratory waterfowl. It is monophyletic, defined by the homoplastic mutations at sites T244C and C588T, along with the amino acid substitution at NA:S82P. The D1.1 and D1.3 PB2 gene tree shows the single monophyletic outbreak clade defined by six nucleotide mutations, including the unique mutation at site C2082T and the five convergent mutations at G726A, G879A, G1086A, G1272A, and A1707G.

## 4. Discussion

For over a decade, public health experts have warned of the eventuality of another influenza pandemic [26]. With the rapid evolution in recent history and the ongoing detection in dairy cattle in the U.S., HPAI A(H5N1) is being actively monitored for pandemic potential [27]. The CDC’s 2024–2025 avian influenza A(H5N1) emergency response demonstrated how the U.S. is poised to act during an evolving outbreak by generating rapid, high-quality genomic data, which is paramount for informing public health authorities, determining risk assessment, and ensuring that existing diagnostics and therapeutics remain effective. By incorporating diverse laboratory methods and sequencing technologies, CDC’s response effort produced high confidence influenza genomic data and analyses that were publicly shared within days of sample collection. Combining these approaches also produced data for a variety of sample types and qualities, including samples with viral loads typically considered too low for routine NGS surveillance.

### 4.1. Turnaround Timing

As active surveillance was essential during the response, epidemiologists were deployed to sites with infected dairy herds or poultry farms to test those directly exposed through their work and monitor contacts of anyone who tested positive for A(H5) influenza. Further testing was also performed for influenza A cases that were unsubtypeable by seasonal influenza diagnostic panels. The CDC coordinated with state public health laboratories across the U.S. to ensure rapid testing of all potential A(H5N1) cases. Once a potential case was identified and the CDC received the specimen, case positivity results and a preliminary genome assembly were typically available within 24 h and the results were communicated with the originating state public health laboratory. Within 48 h, NGS data would routinely be ready for public database submission. In some cases, when the sample had low viral load or was degraded, the timeline to public data release was extended to account for confirmatory diagnostic testing, resequencing of the specimen, or to attempt a targeted sequencing approach (Figure 1).

Through the close coordination of state public health laboratories, health care professionals, and the CDC, A(H5N1) cases were diagnosed, characterized, described, and reported with high confidence, driving public health decision-making and assessment in real time. This coordination empowered health officials to connect with close contacts of positive cases that would benefit from targeted surveillance to ensure early intervention if needed. Throughout the response, no contacts contracted an A(H5N1) infection, further supporting the conclusion that human-to-human transmission was unlikely to have occurred during this outbreak.

### 4.2. Benefits of Deep Sequencing and Replicate Sequencing

The first identified human case of A(H5N1) in this outbreak had a Ct value of <20, which was easily sequenced in triplicate using a typical influenza workflow. In contrast, the second identified case had Ct values greater than 33 for influenza A and H5 diagnostic targets. As this was a positive case associated with a different population, sequencing the HA and NA of this virus was of high interest. However, due to the high Ct values being outside the threshold for routine influenza surveillance sequencing methods, this case spurred the development of targeted methods to gain as much sequencing data as possible for similar future cases. Over the course of the A(H5N1) response, the sequencing workflow was updated to regularly include targeted amplification, deep sequencing, and technical laboratory replicates, allowing for more consistent genome assemblies for specimens with Ct values outside the range for sequencing. Additionally, the primers for targeted amplification of the HA and NA gene segments were updated throughout the response to improve the amplification of the sequences that were currently circulating in the outbreaks. This framework allowed for rapid adjustments to sample processing and was necessary for responding to this public health incident.

### 4.3. Limitations and Observations During the Response

As specimens were sequenced using multiple replicates and sequencing methods, the consensus viral genome sequences within a specimen were compared and were typically found to be identical. The bases that were most likely to differ between methods or to have consistent minor variants were those within primer-binding sites. This can be clearly seen through the HA and NA gene specific primers that were designed with mixed sites. In the consensus assemblies, there is also a consistently called variable site at base 1050 in the HA gene, which is an artifact of the whole genome amplification primers used in this study, which appear to have been binding off-target at this region in A(H5) HA segments.

More minor variant sites were also seen in genome assemblies when sequences were generated from targeted enrichment methods. When targeted enrichment was used, further analysis was not completed on the minor variant sites due to the high potential of variation introduced during laboratory procedures. Targeted enrichment was only used on samples with very high Ct values and on degraded samples, which were amplified prior to library preparation and enrichment. The PCR cycles were kept to a minimum; however, introducing extra amplification steps during the enrichment procedure created more opportunities for introducing minor variants with artificial detection frequencies.

While the confidence in the minor variant data decreased with the original specimen quality, the confidence in the influenza viral gene segments consensus assemblies remained high. During an outbreak response, tracking minor variants can be a useful tool to identify, for example, intra-host viral diversity and adaptation, but having confidence in the consensus genome assembly has a more immediate impact. While there was a clear trade-off during the CDC’s A(H5N1) response, producing high-quality influenza genome assemblies was the priority.

### 4.4. Phylogenetics

As these influenza A(H5N1) viruses primarily circulated in animal hosts, incorporating external data from USDA enhanced phylogenetic results by contextualizing human cases alongside samples collected from animals was also performed. Clustering observed in trees supported transmission events from animals to humans, which aligned with epidemiological data describing the settings of exposure among cases [3]. Without comparable sequence data from animal samples, the resulting phylogenetic trees may have appeared to suggest human-to-human transmission.

Leveraging multiple public sequence data and metadata repositories (GISAID EpiFlu, NCBI GenBank, NCBI Biosample, and NCBI SRA) was also essential for aggregating and deduplicating influenza viral genomic data with location, host, and time metadata. Public data sharing continues to be crucial for situational awareness and One Health-driven characterization of multi-host disease outbreaks. Publishing time and location metadata also informs transmission analyses and is necessary for accurately estimating divergence times between related viral gene segments. Densely sampled viral genomic data is rendered largely useless without proper metadata contextualization.

## 5. Conclusions

Consistent innovations in NGS technologies will continue to improve the speed and accuracy of data, especially during public health responses like the CDC’s 2024–2025 avian influenza A(H5N1) response in the U.S. This response has also highlighted the need for innovation in current influenza sequencing protocols, especially regarding clinical samples of non-seasonal viruses that may have low viral loads, or, in the case of A(H5) in the U.S., which necessitated the use of non-respiratory specimens for case identification and downstream characterization.

Outside of the laboratory methods, the ability for rapid and reproducible analysis was highly valuable to the turnaround time of public data release. The MIRA-NF pipeline was used for rapid analysis for ONT runs. For Illumina sequencing, the in-house CDC analysis pipeline assembled data and then applied QC measures as soon as a sequencing run was completed. The current iteration of this pipeline has been shared for years with National Influenza Reference Centers and Influenza Sequencing Centers, and MIRA-NF is in the process of including the QC metrics for public availability.

As influenza A(H5N1) viruses continue to circulate globally in wild birds, the U.S. CDC remains poised for rapid deployment of comprehensive scientific methods and analyses, ensuring that public health stakeholders benefit from thorough, accurate, real-time situational awareness.

## Figures and Tables

**Table 1:** Influenza-specific primers for CDC’s 2024–2025 A(H5N1) emergency response in the U.S.

Primer ID	Segment Amplification	Sequence (5′-3′) ^	Nucleotide Position *
Uni12/Inf-1	Genome	GGG GGG AGC AAA AGC AGG	5′ UTR
Uni12/Inf-3	Genome	GGG GGG AGC GAA AGC AGG	5′ UTR
Uni13/Inf-1	Genome	CGG GTT ATT AGT AGA AAC AAG G	3′ UTR
H5_HA_F23M	HA	GTC AAA ATG GAG AAM ATA GTR CTW CT	23–48
H5_HA_R1000m	HA	TGA TTT CAC RTA TTT GGG RCA TTC	1000–977
H5_HA_F810Y	HA	YAA AAT TGT CAA GAA RGG RGA CTC	838–861
H5_HA_R1773	HA	CAA GGG TGT TTT TAA CTA CAA TCT G	1768–1744
H5N1_NA_F18	NA	AAA ATG AAT CCA AAT CAA AAG ATA ACA AC	18–46
H5N1_NA_R856	NA	CRG CAT CAG GAT AAC AGG AGC A	876–855
H5N1_NA_F638	NA	CAG ACA CYA TCA AGA GYT GGA GGA A	658–682
H5N1_NA_R1444	NA	GTT TTT TGR ACA AAC TAC TTG TCA ATG GT	1444–1416

^ Binding bases are underlined. * Nucleotide position of HA and NA primers are presented according to A/dairy_cow/Texas/24-029328-01/2024_H5N1 (EPI_ISL_19088565).

## Data Availability

The assembled sequencing data presented in this study are available in NCBI GenBank or in GISAID databases; all accession IDs are included in Supplemental Table S2. The assemblies are also available through the GISAID EpiSet under: EPI_SET_260213up. MIRA-NF analysis workflow is available at https://github.com/CDCgov/MIRA-NF (accessed on 1 August 2025), while the in-house analysis pipeline is not publicly available due to private infrastructure referenced throughout it. However, all quality control measures that are used throughout the analysis are defined in this paper for replication. The code for acquiring USDA NGS data and its associated metadata is available on GitHub at https://github.com/CDCgov/NCBIH5N1MetadataParser (v1), (accessed on 1 August 2025). The code for recreating the phylogenetic analysis is also available on GitHub at https://github.com/CDCGov/HumanH5N1Phylogenetics (v1), (accessed on 1 August 2025). The GISAID data analyzed in this study is indexed as an EpiSet under ID: EPI_SET_260212nr (https://doi.org/10.55876/gis8.260212nr).

## Supplementary Materials

The following supporting information can be downloaded at: https://www.mdpi.com/article/10.3390/v18040482/s1, Supplement Table S1. All influenza-specific primers used during the CDC’s 2024–2025 influenza A(H5N1) emergency response in the U.S.; Supplemental Table S2. A(H5N1) sequences released to public databases with sequencing method and accession ID; Supplemental Table S3. Summary of A(H5)-positive specimens: Ct values and sequencing success; Supplemental File S1: Nextstrain builds for HA, NA, and PB2; Supplemental Figure S1: A(H5N1) case flow through to public database release.

## Abbreviations

The following abbreviations are used in this manuscript:

NGS	next-generation sequencing
HPAI	highly pathogenic avian influenza
CDC	Centers for Disease Control and Prevention
HA	Hemagglutinin
NA	Neuraminidase
ONT	Oxford Nanopore Technologies
USDA	U.S. Department of Agriculture
NVSL	National Veterinary Services Laboratory
QC	quality control
SNV	single-nucleotide variants
MAF	minor allele frequency
SRA	NCBI’s Sequence Read Archive
GISAID	Global Initiative for Sharing All Influenza Data
LPAI	low pathogenic avian influenza
CS	conjunctival swab
NP	nasopharyngeal swab
NS	nasal swab
OP	oropharyngeal swab
BAL	bronchoalveolar lavage sample
InfA	influenza A
bp	base pair
PB2	polymerase basic protein 2
UTR	untranslated region
LOD	limit of detection
NUC	nucleotide
