# Supplementary figures and images for: Next-Generation Sequencing Strategies During the 2024–2025 Avian Influenza A(H5N1) Emergency Response in the U.S
Source: Viruses. 2026 Apr 21;18(4):482. doi: 10.3390/v18040482 (PMC13120661; doi:10.3390/v18040482)

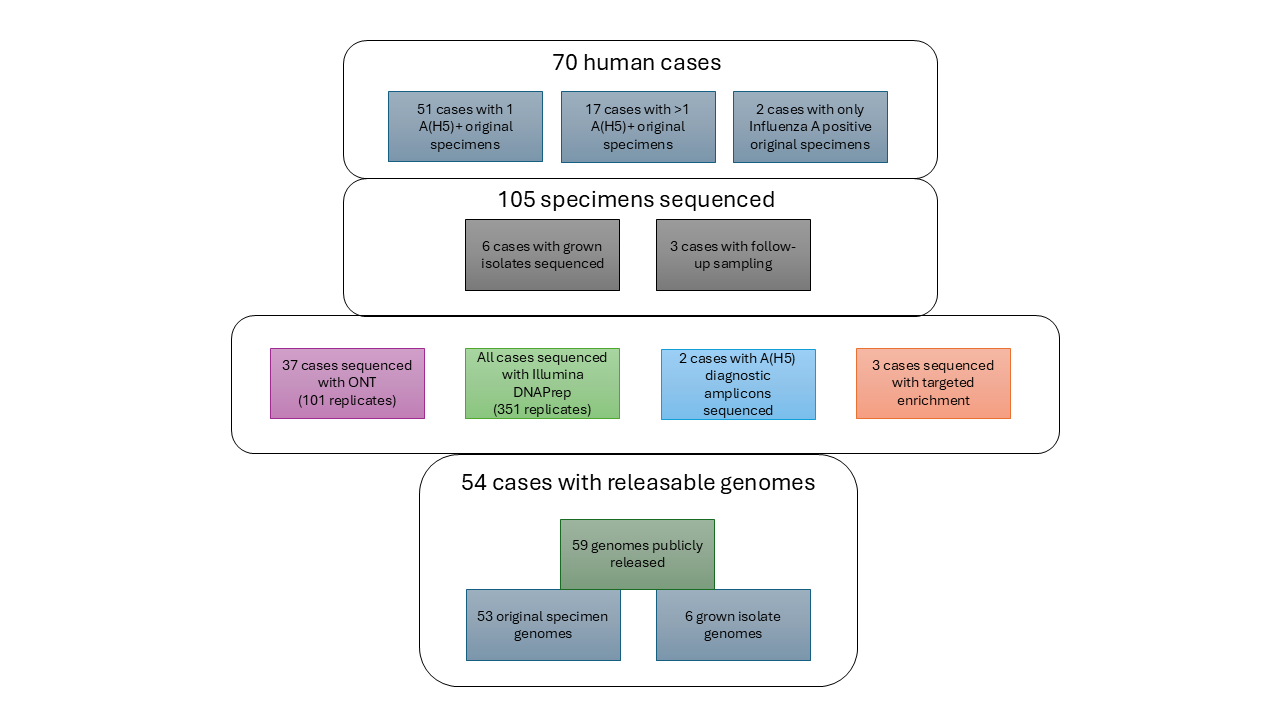

Supplement: Supplementary file 1 [file viruses-18-00482-s001.zip › SupplementalFigureS1_AH5N1-case-flow-through-to-public-database-release.png]
